# The Rheology and Printability of Cartilage Matrix-Only Biomaterials

**DOI:** 10.3390/biom12060846

**Published:** 2022-06-17

**Authors:** Emi A. Kiyotake, Michael E. Cheng, Emily E. Thomas, Michael S. Detamore

**Affiliations:** 1 Stephenson School of Biomedical Engineering, University of Oklahoma, Norman, OK 73019, USA; emi.kiyotake@ou.edu (E.A.K.); michael.cheng@ou.edu (M.E.C.); 2 Department of Biomedical Engineering, University of Michigan, Ann Arbor, MI 48109, USA; emilyet@umich.edu

**Keywords:** cartilage, hydrogels, rheology, methacryloylation, extracellular matrix, bioprinting

## Abstract

The potential chondroinductivity from cartilage matrix makes it promising for cartilage repair; however, cartilage matrix-based hydrogels developed thus far have failed to match the mechanical performance of native cartilage or be bioprinted without adding polymers for reinforcement. There is a need for cartilage matrix-based hydrogels with robust mechanical performance and paste-like precursor rheology for bioprinting/enhanced surgical placement. In the current study, our goals were to increase hydrogel stiffness and develop the paste-like precursor/printability of our methacryl-modified solubilized and devitalized cartilage (MeSDVC) hydrogels. We compared two methacryloylating reagents, methacrylic anhydride (MA) and glycidyl methacrylate (GM), and varied the molar excess (ME) of MA from 2 to 20. The MA-modified MeSDVCs had greater methacryloylation than GM-modified MeSDVC (20 ME). While GM and most of the MA hydrogel precursors exhibited paste-like rheology, the 2 ME MA and GM MeSDVCs had the best printability (i.e., shape fidelity, filament collapse). After crosslinking, the 2 ME MA MeSDVC had the highest stiffness (1.55 ± 0.23 MPa), approaching the modulus of native cartilage, and supported the viability/adhesion of seeded cells for 15 days. Overall, the MA (2 ME) improved methacryloylation, hydrogel stiffness, and printability, resulting in a stand-alone MeSDVC printable biomaterial. The MeSDVC has potential as a future bioink and has future clinical relevance for cartilage repair.

## 1. Introduction

Cartilage extracellular matrix (ECM) contains bioactive signals that guide cell adhesion, proliferation, and differentiation, and is thought to be chondroinductive and promising for cartilage repair after cartilage injury or osteoarthritis [1]. Across cartilage matrix-based materials developed to date, the field is missing cartilage matrix-based materials that are (1) bioprintable/easy to surgically deliver and (2) mechanically robust (e.g., have high elastic moduli and failure strain akin to native cartilage). In terms of mechanical performance, native human articular cartilage has a compressive elastic modulus of about 1.8 MPa [2], which is an order of magnitude higher than most cartilage matrix-based materials, and higher than many nanocomposite hydrogels. Nanocomposite hydrogels for cartilage repair, but not made with cartilage matrix, which have added nanomaterials for mechanical reinforcement (e.g., carbon-based, ceramic, metallic, polymeric), typically have compressive elastic moduli less than 1 MPa [3,4,5,6,7,8]. Cartilage matrix-based materials typically have compressive elastic moduli less than 160 kPa, even with additional crosslinking (e.g., carbodiimide, dehydrothermal treatment, genipin, UV) or with added hydrogel components (e.g., alginate, collagen I, gelatin methacrylate (GelMA)) [9,10,11,12,13,14,15,16,17,18,19]. Cartilage matrix-containing materials that achieved elastic moduli greater than 200 kPa have been mechanically reinforced with plastic support scaffolds (e.g., polycaprolactone (PCL), poly(lactic-co-glycolic acid) (PLGA)) [20,21,22]. While mechanical robustness has been achieved with plastic support scaffolds, the surgical delivery of prefabricated scaffolds may be more challenging (e.g., removal of healthy cartilage to fit the scaffold shape). Photocrosslinking hydrogels that act as carrier materials for cartilage particles and can form in situ are promising in terms of easier surgical deliveries (e.g., injectable). However, in addition to cartilage-based hydrogels typically having poor mechanical performance (i.e., <160 kPa compressive moduli), hydrogels have poor retainment after injection because the hydrogel precursors are typically low-viscosity liquids that are prone to leaking out from the defect after the injection before they can crosslink. One solution is to develop cartilage matrix-based hydrogel precursors that are paste-like in consistency to prevent leaking and enable easier surgical delivery.

Fortunately, we can leverage the knowledge from the bioprinting field to develop the paste-like rheology of printable materials for extrusion bioprinting and apply this knowledge to the development of paste-like biomaterials for easier surgical placement. A few pioneering studies have developed cartilage matrix-based printable biomaterials [15,21,23,24]; however, those studies required additional polymers (i.e., alginate, gelatin, GelMA, hyaluronic acid (HA)) to increase the viscosity of the precursor solution for successful bioprinting or plastic support scaffolds (i.e., PCL) to then infill with the cartilage matrix. Overall, there is a need for cartilage matrix-based materials to be developed with paste-like precursors for extrusion bioprinting and easier surgical delivery, and high mechanical performance post-crosslinking. To simultaneously overcome the aforementioned challenges, we previously developed a methacrylated cartilage matrix hydrogel that achieved compressive moduli of ~0.675–1 MPa, functionalized with glycidyl methacrylate (GM) [2,25]. The hydrogel precursor was a photocrosslinkable paste that could be crosslinked in situ with UV light (~10 min crosslinking time), which enabled easier surgical placement. While the compressive modulus approached that of native cartilage, the decellularized version of the MeSDVC hydrogels had low failure strains (~7.5% failure strain), and bioprintability was not considered.

In the current study, we investigated a more common methacrylating reagent, methacrylic anhydride (MA), to increase the functionalization of the solubilized cartilage matrix and compare it to our previous cartilage matrix hydrogels. The purpose of the study was to (1) improve the solid mechanics (i.e., stiffness, failure strain) of the crosslinked cartilage hydrogel to better mimic native cartilage, and (2) refine the fluid mechanics of the hydrogel precursor to obtain paste-like rheology for extrusion printing and in the future, easier surgical delivery.

## 2. Materials and Methods

### 2.1. Materials

Hydrochloric acid (HCl, 37%; HX0603-75), pepsin (^3^2500 units/mg; P7012), N,N-dimethylformamide (DMF, ^3^99.8%; 319937), methacrylic anhydride (94%; 276685), triethylamine (TEA, ^3^99%; T0886), tetrabutylammonium bromide (TBAB, ^3^98%; 426288), glycidyl methacrylate (GM, ^3^97%; 779342), acetone (^3^99.5%; AX0116-1), 3-(Trimethylsilyl)propionic-2,2,3,3-d_4_ acid sodium salt (TMSP; 269913-1G), phosphate-buffered saline (PBS; P3813), and 4′,6-diamidino-2-phenylindole (DAPI; D9542) were purchased from Sigma Aldrich (St. Louis, MO, USA). Sodium hydroxide (NaOH; ^3^97.0%; BDH9292) was purchased from VWR Chemicals BDH^®^ (Radnor, PA, USA). Deuterium oxide (D_2_O; DLM-4-100) was purchased from Cambridge Isotope Laboratories, Inc., (Andover, MA, USA). Lithium phenyl-2,4,6-trimethylbenzoylphosphinate (LAP, ^3^98%; TCL0290-1G) was purchased from TCI America (Portland, OR, USA). Pluronic™ F-127 (PF-127, poloxamer 407; PLU-100) was purchased from Allevi (Philadelphia, PA, USA). Antibiotic-antimycotic (AA; 15240062), minimal essential medium a (a-MEM; 12561072), fetal bovine serum FBS; certified, Performance Plus, 16000044), penicillin/streptomycin (P/S; 15140122), and trypsin (25200072) were purchased from Fisher. Fibroblast growth factor-2 (FGF-2; 100-18B) was purchased from PeproTech (Cranbury, NJ, USA).

### 2.2. Synthesis of Methacryl-Modified Solubilized Devitalized Cartilage

**Figure 1:**
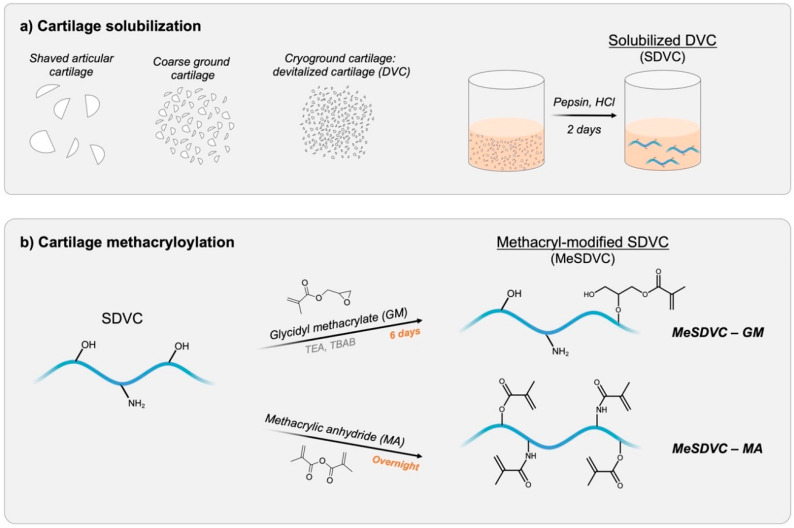
Illustration of cartilage processing and functionalization. (**a**) Devitalized cartilage (DVC) particles were formed by shaving porcine articular cartilage from the knee, freezing, coarse-grinding with dry ice, and cryogrinding into smaller particles using a cryogenic tissue grinder to form devitalized cartilage (DVC). To form solubilized DVC (SDVC), the DVC particles were solubilized with pepsin in hydrochloric acid (HCl) for 2 days, dialyzed, and lyophilized. (**b**) SDVC was methacryloylated with either (1) glycidyl methacrylate (GM) plus triethylamine (TEA) and tetrabutylammonium bromide (TBAB) for 6 days or (2) methacrylic anhydride (MA) overnight. GM functionalizes mostly hydroxyls and carboxyls of the polymers present in the cartilage matrix (e.g., collagens, hyaluronic acid) to form MeSDVC—GM. In addition to hydroxyls and carboxyls, MA additionally functionalizes amines to form MeSDVC—MA.

Cartilage was harvested from 4 porcine wrists (Hampshire and Berkshire, female, 1 year, 180–220 kg), washed in deionized (DI) water, and stored at –20 °C. Devitalized cartilage (DVC) particles and solubilized DVC (SDVC) were formed as previously described (Figure 1a) [2,25,26,27,28]. Briefly, to form coarse ground cartilage, cartilage was thawed, minced, and coarse ground with dry ice in a coffee grinder (Kitchen Aid, Benton Harbor, MI, USA). To form DVC, coarse ground cartilage was then lyophilized and cryoground in a SPEX 6770 Freezer/Mill (SPEX SamplePrep, Metuchen, NJ, USA) with the following settings: 2 min pre-cooling, 2 min run time, 2 min cool time, 10 cycles at a rate of 15 cycles per second (cps). The DVC was lyophilized on a FreeZone 6 Plus lyophilizer (Labconco, Kansas City, MO, USA) and stored at −20 °C.

To form SDVC, DVC (1 g/100 mL) was solubilized in pepsin (1 mg/mL in 0.1 M HCl) for 2 days at room temperature. The SDVC was titrated with NaOH to pH 8–10 and centrifuged (6000× *g*, 3 min) in a 5430R centrifuge (Eppendorf, Hamburg, Germany). The supernatant containing the SDVC was collected and the non-solubilized pellet was discarded. The SDVC in the supernatant was dialyzed (Dialysis Membrane, Standard RC Tubing, MWCO:6–8 kD, 64 mm diameter, Spectra/Por 132670, Los Angeles, CA, USA) against DI water for 2 days with water changes every 12 h, frozen at –20 °C, and lyophilized.

Methacryl-modified SDVC (MeSDVC) was synthesized (Figure 1b) with glycidyl methacrylate, as previously described [25], or with methacrylic anhydride, using a protocol adapted from Tsanaktsidou et al. [29]. To synthesize MeSDVC with glycidyl methacrylate (GM), SDVC (1 g) was dissolved in DI water (112.5 mL), and then acetone (37.5 mL, 1:3 acetone:water ratio) was slowly added. TEA (catalyst, 2.2 mL), GM (2.2 mL), and TBAB (phase transfer catalyst, 2.2 g) were separately mixed, where the GM was added at a 20-fold molar excess to SDVC (molar excess calculation based on previous work [25], where a 20-fold molar excess corresponded to 2.2 mL of GM per g of SDVC). The methacrylation solution was added to the SDVC solution and stirred for 6 days at room temperature. The MeSDVC-GM was precipitated at 8 times the reaction volume of acetone and centrifuged (5430 R centrifuge, 6000× *g*, 3 min). The acetone was decanted, and the pellet of MeSDVC-GM was dissolved in DI water (100 mL), dialyzed against water for 2 days, frozen at –20 °C, lyophilized, and stored at –20 °C.

To synthesize MeSDVC with methacrylic anhydride (MA), SDVC (1 g) was dissolved in DI water (100 mL), and then DMF (100 mL, 1:1 ratio water:DMF) was slowly added. MA was slowly added to the reaction solution in different molar ratios to SDVC: 2-, 5-, 10-, and 20-fold molar excesses, or 0.744, 1.86, 3.72, and 7.44 mL of MA per gram of SDVC, respectively. The pH was maintained between 8 and 9 with NaOH for 1.5 h, or until the pH was stable. The reaction was stirred overnight at room temperature. The MeSDVC-MA was precipitated and processed using the same aforementioned steps as the MeSDVC-GM.

### 2.3. Quantification of Functionalization with NMR

The functionalization of SDVC and each batch of MeSDVC were quantified through nuclear magnetic resonance (NMR), as previously described [30]. Briefly, 1–3 mg of lyophilized samples were dissolved in 0.7 mL of D_2_O with TMSP (1 mg/mL) as an internal standard. Each dissolved sample was loaded into a 5-mm NMR tube (Norell, Morganton, NC) with a glass Pasteur pipette, and ^1^H NMR spectra were collected at 80 °C on a VNMRS-500 MHz NMR Spectrometer equipped with a 5 mm indirect detection room temperature probe (Varian, Palo Alto, CA, USA). To ensure proper relaxation of the functional groups and accurate quantification, the following parameters were used: 16 scans, 35 s recycle delay, 90-degree pulse width, and a 60 s pre-acquisition delay. All spectra were analyzed in MestReNova software v.12.0.1 (Mestrelab Research, Santiago de Compostela, Spain), with a 1 Hz exponential apodization, baseline correction (Bernstein polynomial fit, order 3), phase correction, and referencing the water peak at 4.163 ppm.

The total methacrylation was determined by integrating the signals (5.40–5.65 ppm) corresponding to one proton of the methacrylamide and one proton of methacrylate, with each normalized to the nine protons in the TMSP signal (–0.22 ppm), as done by Claaßen et al. [31]. The degree of functionalization in mmol/g of MeSDVC was quantified by the following equation:

mmol methacryl/g MeSDVC = (∫ methacryl/∫ TMSP) * (9 H/1 H) * (mmol TMSP/g MeSDVC)(1)

To determine the degree of methacrylation, the signals (5.80–6.00 ppm) corresponding to the other methacrylate proton were integrated and calculated (Equation (1)). The degree of methacrylamide functionalization was determined by subtracting the degree of methacrylation from the total methacryloylation.

### 2.4. Hydrogel Crosslinking

**Figure 2:**
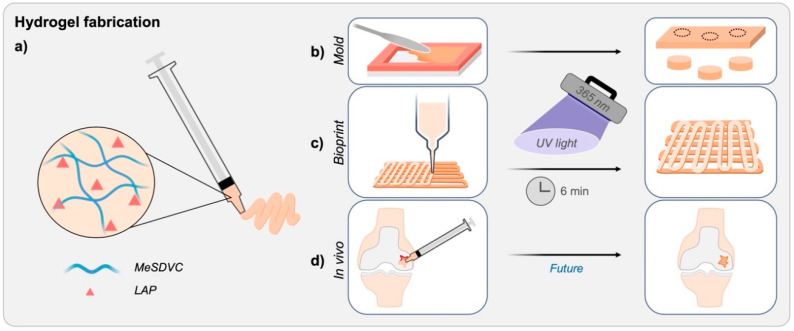
Illustration of the applications of the paste-like cartilage hydrogel precursor and photocrosslinking hydrogel. (**a**) The methacryloyl-functionalized and solubilized devitalized cartilage (MeSDVC) precursor is comprised of only MeSDVC and a photoinitiator, LAP. The paste-like precursor was easily loaded into syringes for ease of application. The paste-like MeSDVC precursors (**b**) were loaded into molds and (**c**) were printed. After exposure to UV light (365 nm) for 6 min, the precursors photocrosslinked into a stiff hydrogel. Hydrogels formed in molds were biopsy-punched out for hydrogel characterization (e.g., swelling, stiffness), and printed structures were characterized (e.g., pore areas and strut size measurements) or seeded with cells. (**d**) In the future, the MeSDVC paste-like precursor may prevent material leakage from a cartilage defect and the quick in situ crosslinking may enable the formation of a high-modulus hydrogel, similar to native cartilage, to support an immediate return to weight-bearing activities.

Hydrogel fabrication for characterization and printing is illustrated in Figure 2. All MeSDVCs were dissolved in PBS (10 *w*/*v*%) containing the photoinitiator, LAP (2.2 mM) (Figure 2a), and mixed well with a spatula. For stiffness and swelling characterization, hydrogel precursors were loaded into a rectangular rubber gasket mold (1 mm thickness) between two glass slides and crosslinked with a handheld UV light (EB-160C, Spectroline, Westbury, NY, USA) with a 365 nm bulb (6–9 mW/cm^2^) for 3 min on each side (6 min total) (Figure 2b). Cylindrical discs were punched out of the formed hydrogel using a 6-mm biopsy punch. For printing, the hydrogel precursors were loaded into a 10 cc syringe barrel and printed with a tapered nozzle (Figure 2c). Printed scaffolds for bioink characterization were not crosslinked, but printed scaffolds for in vitro studies were crosslinked with a 365 nm UV light in a biosafety cabinet for 6 min, only from the top. In the future, the MeSDVC cartilage hydrogels can be applied in vivo (Figure 2d), where the hydrogel loaded into a syringe is injected into a cartilage defect, smoothed in place with a spatula, and crosslinked in situ.

### 2.5. Hydrogel Stiffness and Swelling

Hydrogel stiffness was determined by evaluating the compressive elastic modulus (n = 4–6) of 6-mm punched hydrogels that were swollen in PBS overnight at 37 °C. The diameters of swollen hydrogels were measured with a digital micrometer (293-340-30; Mitutoyo, Kawasaki, Japan). Then, hydrogels were compressed (5 µm/s, ~0.49% strain/s) on a Discovery Hybrid Rheometer-2 (DHR-2; TA Instruments, New Castle, DE, USA) until a 20% strain at 25 °C under dry conditions, as previously described [30,32]. Prior to compression, hydrogels had a 0.1 N tare load to measure the hydrogel height (1.20 ± 0.08 mm) and ensure contact between the geometry and hydrogel. The compressive elastic moduli were calculated as the slope of the linear region of the stress–strain plot (i.e., 5–15% strain).

Swelling and absorption characterizations (n = 3–6) were performed as previously described [30]. The fabricated mass of each hydrogel was measured immediately after crosslinking and being punched, the swollen mass was measured after swelling in PBS overnight at 37 °C, and the dry mass was measured after hydrogels were lyophilized. The absorption was calculated as the swollen mass divided by the fabricated mass. The swelling ratio was calculated as the swollen mass divided by the dry mass.

Images of fabricated and punched hydrogels were taken on a Nikon D5500 with a macro lens, Nikon AF-s Micro-NIKKOR 60 mm f/2.8G ED Lens (B&H Photo Video, New York City, NY, USA).

### 2.6. Hydrogel Precursor Rheology

The yield stress (n = 3–5), storage modulus recovery (n = 5), and viscosity (n = 3–5) of each hydrogel precursor were characterized on a DHR-2 rheometer (TA Instruments) with 20 mm crosshatched plates at 25 °C and a 500 µm gap, as previously described [30,32,33,34]. PF-127 and unfunctionalized SDVC were additionally included as comparator groups. Given that PF-127 is a commonly used printable support material, rheology on PF-127 was included to provide a reference point. The yield stress was determined using the modulus crossover method, which is the measured stress (x-value) at which the storage and loss moduli cross during an oscillatory shear stress sweep (1–3000 Pa) [30,35]. The storage modulus recovery was determined as the percent of the storage modulus recovered ~5 s after shearing compared to its baseline, which was tested using three steps of oscillatory shearing (1 Hz) after a 1 min soak time: 1 min of 10 Pa stress (baseline), 30 s of 3000 Pa stress (shearing the sample beyond its yield stress), and 2 min of 10 Pa stress (recovery). The viscosities were measured during a logarithmic shear rate sweep (0.1–100 s^−1^). Images of ~200 µL of hydrogel precursors were taken on a Nikon D5500 with a macro lens.

### 2.7. Bioink Characterization

For printing, the hydrogel precursors were backloaded into a syringe barrel (10 cc, 7012112; Nordson EFD, East Providence, RI, USA), and centrifuged in a 5920R centrifuge (Eppendorf) for 20–30 s to eliminate bubbles before printing. The hydrogel precursors were printed on a BioAssemblyBot^®^ 400 (Advanced Solutions, Louisville, KY, USA) through a tapered plastic nozzle (22 G, TE-TT22-DHUV; OK International, Cypress, CA, USA). All images and videos were taken on a Nikon D5500 with a macro lens.

To qualitatively assess the shape fidelity of the prints, hydrogel precursors, SDVC, and PF-127 were printed (n = 3–8) at room temperature into 4-layer square grids (9.68 × 9.68 × 0.94 mm, L × W × H) with the printing parameters listed in Table 1. The pore areas and strut sizes were measured as previously described [30,34]. The pore areas and the number of pores for each print were calculated from the images using ImageJ software (National Institutes of Health, Bethesda, MD, USA) and the Analyze Particles feature. The strut size for each print was measured and averaged in ImageJ from 3 horizontal and 3 vertical struts in each print. Given that the pore size and strut size may be controlled by altering the pressure, we adjusted the pressure for each material to obtain the best print possible (see Table 1 for the pressures and printing parameters used for the materials). For the filament collapse test (n = 3–6), the hydrogel precursors were printed as a 54 mm line across gaps of varying distances (i.e., 1, 2, 4, 8, 16 mm), as previously described [30,36,37], with modifications to the analysis. Specifically, the printing was recorded and the lowest height of the filament in each gap was analyzed 20 s post-print (i.e., after the filament stopped moving). To qualitatively assess the structural integrity of taller prints, the MeSDVC 2 ME hydrogel precursor was printed into an 8-layer grid (9.68 × 9.68 × 1.94 mm, L × W × H).

### 2.8. rBMSC Harvest and Culture

The rBMSCs were harvested (IACUC protocol no. R20-001) from the femurs of male CD^®^ Sprague Dawley rats (252–275 g; Charles River Laboratories, Wilmington, MA, USA), as previously described [25,28]. Rats were humanely euthanized according to the American Veterinary Medical Association (AVMA) guidelines by CO_2_ inhalation. The hind legs were removed and put in PBS with 1% antibiotic-antimycotic (AA) until transferred to a biosafety cabinet. The femur was detached from the tibia and fibula by opening up the knee joint, and the muscle and tendons were removed from the femur with a no. 10 scalpel blade and rinsed with PBS + 1% AA. Each end of the femur was cut off with surgical scissors (6.75” Mayo Straight Dissecting Blunt, Premium Instruments, Amazon, Seattle, WA, USA), and the bone marrow was flushed out with 1 mL of a-MEM with 10% fetal bovine serum (FBS) and 1% AA into a sterile 1.7 mL tube using a 3-mL syringe with a tapered plastic nozzle (18 G, TT18-DHUV-PK; OK International). A new 3-mL syringe with a needle (18 G) was used to break up the bone marrow and transfer it to a T-25 tissue culture treated flask (TP90026, MIDSCI, Valley Park, MO, USA) with 5 mL of a-MEM (10% FBS, 1% AA). The cells were left to adhere for 24 h before being rinsed with PBS and then cultured with a-MEM (10% FBS, 1% penicillin/streptomycin (P/S), 2 ng/mL of fibroblast growth factor-2 (FGF-2). Medium changes were done every 2–3 days and the rBMSCs were passaged when 80% confluent using trypsin.

### 2.9. Printed In Vitro Cell Viability Study

The lyophilized MeSDVC 2 ME was sterilized with ethylene oxide gas (AN74i, Anderson Anprolene, Haw River, NC, USA) before being prepared and loaded into syringe barrels as described in the Hydrogel Crosslinking and Bioink Characterization sections. The MeSDVC was printed into 4-layer grids (9.68 × 9.68 × 0.94 mm, L × W × H) in each well of 12-well plates (print parameters: 0.3 mm layer height, 200 ms start delay, 9 psi, 22 G tapered nozzle) on a BioAssemblyBot under sterile conditions at room temperature. Printed MeSDVC grids in the 12-well plates were transferred to a biosafety cabinet and were crosslinked for 6 min using a handheld UV light (365 nm). The rBMSCs (Passage 2) were seeded on the crosslinked–printed MeSDVC hydrogels (100,000 cells/scaffold). The rBMSCs were cultured for 15 days in the same medium as for expansion (a-MEM, 10% FBS, 1% P/S, 2 ng/mL FGF-2) and medium changes were done every 3 days. The printed scaffolds were 4 layers tall, and during seeding, the cells were noted to have not settled on the top-most layer of printed struts, but instead settled on the second-from-the-top layer of struts that were exposed, before adhering.

On days 3 and 15, MeSDVC hydrogels (n = 3) were stained with the LIVE/DEAD^®^ Viability/Cytotoxicity Kit for Mammalian Cells (Thermo Fisher Scientific, L3224) or with Phalloidin-iFluor 488 Reagent (ab176753, Abcam, Boston, MA, USA) for F-actin (n = 3). For live/dead staining, hydrogels were rinsed with PBS and stained for 20 min with calcein AM (2 µM) and ethidium homodimer-1 (4 µM), according to the manufacturer’s instructions. The hydrogels for F-actin staining were stained according to the manufacturer’s protocols. Briefly, MeSDVC hydrogels were rinsed with PBS, fixed in 10% formalin (VWR 89370-094) for 20 min, rinsed 3 times in PBS, permeabilized with Triton X-100 (0.1%, Sigma T8787) for 20 min, stained with a 1X Phalloidin conjugate working solution with DAPI (500 nM) for 1 h at room temperature, and rinsed 3 times with PBS.

Hydrogels were imaged on a Leica TCS SP8 confocal laser scanning microscope (Leica Microsystems, Wetzlar, Germany). The entire scaffold was scanned with Tile Scan using a 5× objective with 0.15 NA (12–15 tiles). Sample regions were sequentially scanned with a 20× objective with 0.75 NA to acquire z-stacks. The argon laser was used to excite the calcein AM for live-cell signals with the emission detection window set to 500–550 nm. The DPSS 561 laser was used to excite ethidium homodimer-1 to detect the dead cell signals with the emission window set at 570–700 nm. The 405 Diode laser and DPSS 561 laser were used to excite the DAPI and phalloidin, respectively. Emission detection windows for DAPI and phalloidin were set to 415–470 nm and 500–550 nm, respectively. The tiles were merged using the Leica LAS X software with the linear blending algorithm and the smooth overlap. Channels and z-series were merged using ImageJ software.

### 2.10. Statistical Analyses

GraphPad Prism version 9.3.1 for macOS (GraphPad Software, San Diego, CA, USA, www.graphpad.com) was used to perform all statistical analyses. A one-way ANOVA with Tukey’s post hoc test was used to analyze the compressive modulus, absorption, swelling, yield stress, storage modulus recovery, and pore/strut size data. A two-way ANOVA with Tukey’s post hoc test was used to analyze the viscosity and filament collapse height data. Significance was considered at a level of *p* < 0.05. All results were reported as mean ± standard deviation.

## 3. Results

### 3.1. NMR Quantification of Functionalization

**Figure 3:**
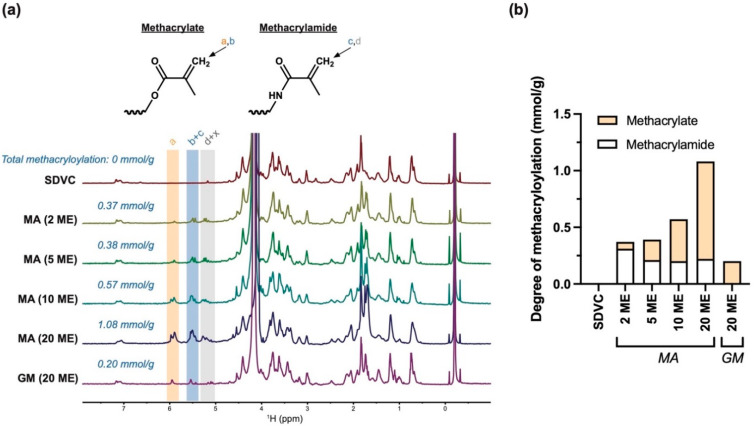
Solubilized cartilage functionalized with methacrylic anhydride had higher functionalization than glycidyl methacrylate. (**a**) The structures of methacrylates (i.e., functionalized hydroxyls/carboxyls) and methacrylamides (i.e., functionalized amines) are shown with the two CH_2_ protons of methacrylates labeled as “a” and “b” and the two CH_2_ protons of methacrylamides labeled as “c” and “d”, which correspond to the highlighted portions of the NMR spectra. The NMR spectra of SDVC and MeSDVC functionalized with different molar excesses (ME) of methacrylic anhydride (MA) (i.e., 2, 5, 10, 20 ME) and 20 ME of glycidyl methacrylate (GM) are shown. Total methacryloylation (in blue text) was determined via integration of the signals arising from one proton of the methacrylate and one proton of the methacrylamide (i.e., 5.40–5.65 ppm “b + c”, blue highlight), with normalization to the TMSP internal standard (–0.22 ppm). With increasing molar excesses of MA, there was increased total methacryloylation. (**b**) The proportion of methacrylate and methacrylamide content for each MeSDVC is shown. Methacrylate content was determined via integration of the signals from one proton of the methacrylate (i.e., 5.80–6.00 ppm, “a”, orange highlight). Methacrylamide content was calculated as the methacrylation subtracted from the total methacryloylation. Methacrylamide content was constant across the MA functionalized MeSDVC and the methacrylate content increased with an increased molar excess of MA. The GM did not have methacrylamide signals and did not appear to have functionalized amines.

Given that hydroxyls and amines may both be functionalized, the total methacryloylation of each batch of MeSDVC includes methacrylates (functionalized hydroxyls) and methacrylamides (functionalized amines). The total methacryloylation was quantified via integration of the signals between 5.40 and 5.65 ppm (total methacryloylation is shown in italicized blue text for each MeSDVC batch in Figure 3a), which arose from one proton of the methacrylate CH_2_ (Figure 3a, labeled as “b” in blue on the chemical structures) and one proton of the methacrylamide CH_2_ (Figure 3a, labeled as “c” in blue on the chemical structures). All batches of MA, even the lowest molar excess of 2 ME, had higher total methacryloyl content than that of the 20 ME of GM.

The methacrylate content was quantified via integration of the signals between 5.80 and 6.00 ppm, which was from the other proton of the methacrylate CH_2_ (Figure 3a, labeled as “a” in orange on the chemical structure). The signals between 5.0 and 5.4 ppm included the other proton of the methacrylamide CH_2_ (Figure 3a, labeled as “d” in gray on the chemical structure) and possibly contained additional peaks (e.g., functionalized hydroxyproline [31], labeled as “x” on the NMR spectra). The additional peaks do not enable direct quantification of methacrylamide via integration; therefore, methacrylamide content was instead calculated as the methacrylate content subtracted from the total methacryloyl content. The methacrylate and methacrylamide contents are shown in Figure 3b. While all the MA MeSDVC batches had both methacrylamides and methacrylates, interestingly, the 20 ME GM contained only methacrylates and no detectable methacrylamides. With increasing MA, there was a trend of increased methacrylates, while the amount of methacrylamides was approximately the same.

### 3.2. Hydrogel Stiffness and Swelling

**Figure 4:**
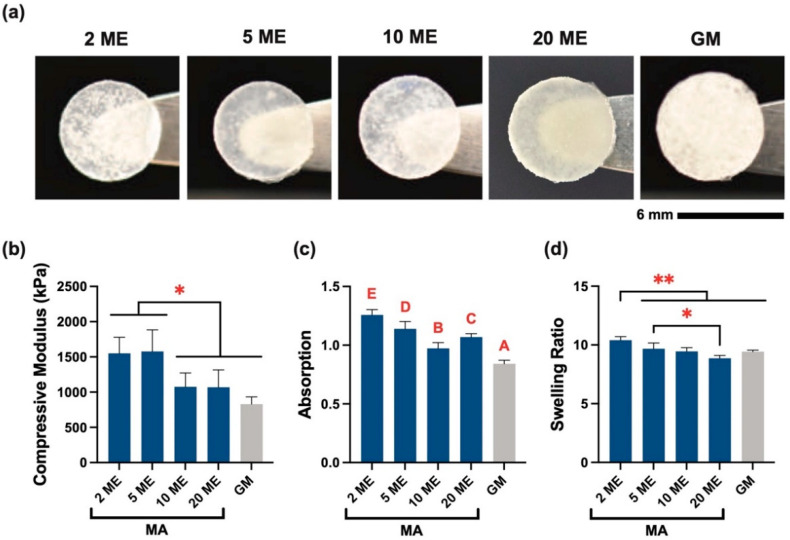
MeSDVC hydrogels had a stiffness close to native cartilage and minimal swelling/contraction. (**a**) The punched-out hydrogels of UV crosslinked MeSDVC hydrogels are shown. There was slight opacity but minimal color differences across groups. (**b**) Interestingly, the lowest functionalized MA MeSDVC (2 and 5 ME) had the highest compressive moduli (1550 ± 230 kPa and 1580 ± 310 kPa, respectively) (n = 4–6), which is close to that of native articular cartilage (~1800 kPa). (**c**) The absorption of the MeSDVC hydrogels was close to 1.0 (ranging between 0.84 ± 0.03 and 1.26 ± 0.05) (n = 3–6) and had minimal diameter changes. Absorption ratios close to 1 indicated minimal water absorption after fabrication, which has advantages in material retention in the defect as the hydrogels may not contract or swell out. (**d**) The swelling ratios were in the range of 8.87 ± 0.24 to 10.41 ± 0.30 (n = 3–6). Scale bar: 6 mm. * *p* < 0.05. ** *p* < 0.01. Different letters indicate significance from each other (*p* < 0.05).

Figure 4a shows representative crosslinked hydrogels for each batch of MeSDVC. The compressive moduli (Figure 4b) of the 2 ME and 5 ME hydrogels (1.55 ± 0.23 MPa and 1.58 ± 0.31 MPa, respectively) were 44% to 90% greater than those of the 10 ME, 20 ME, and GM groups (*p* < 0.05). The 2 ME and 5 ME hydrogels did not have significantly different moduli. All MeSDVC hydrogel groups were able to reach 20% strain without failure (stress–strain curves not shown).

For hydrogel absorption (Figure 4c), the GM MeSDVC hydrogel and 10 ME hydrogel made with MA had water absorption ratios less than 1.0 (i.e., contraction). On the other hand, the 2 ME, 5 ME, and 20 ME hydrogels had minimal water absorption mean values, ranging from 1.07 to 1.26. The 2 ME and 5 ME hydrogels had 6–49% greater absorption than those of the 10 ME, 20 ME, and GM hydrogels, respectively (*p* < 0.05). The 20 ME had 10 and 27% greater absorption than those of the 10 ME and GM hydrogels, respectively (*p* < 0.01). The 10 ME hydrogels had 16% greater absorption than that of the GM hydrogels (*p* < 0.001). All the MeSDVC absorptions were close to 1, indicating minimal hydrogel contraction or swelling after fabrication, which was further evidenced by minimal changes in the hydrogel diameter from fabrication (punched at 6 mm) to the swollen state (MeSDVC diameter range: 5.96 ± 0.10 to 6.06 ± 0.10 mm).

The swelling ratios of all MeSDVC hydrogels (Figure 4d) were in the range of 8.87 ± 0.24 to 10.41 ± 0.30. The 2 ME hydrogels had 7 to 17% greater swelling ratios than those of all of the other hydrogels (*p* < 0.01). The 5 ME had a 9% greater swelling ratio than that of the 20 ME hydrogels (*p* < 0.05).

### 3.3. Hydrogel Precursor Rheology

**Figure 5:**
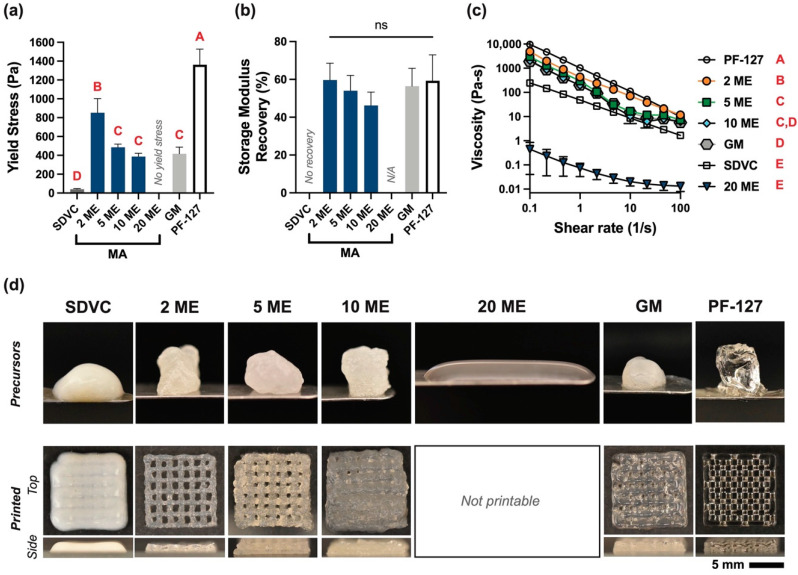
Most MeSDVCs had paste-like precursors, with the 2-fold molar excess MA and GM MeSDVCs printing the best grids. The MeSDVC precursors, along with unfunctionalized SDVC and Pluronic™ F-127 (PF-127) for comparison, were characterized with three rheological tests: (**a**) yield stress, (**b**) storage modulus recovery, and (**c**) viscosity. The 2 ME MA MeSDVC precursor had a greater yield stress and viscosity than those of all other MeSDVCs and SDVCs. Interestingly, the 20 ME MA MeSDVC did not have a detectable yield stress and had significantly lower viscosity than all other groups, which we speculate may have been due to the higher functionalization preventing collagens present in the MeSDVC from forming triple helices. (**d**) The paste-like precursors of MeSDVC, SDVC, and PF-127 are visualized on spatulas (top row) and were printed into four-layer grids to assess printability (bottom row). The 5 ME and 10 ME of MA MeSDVCs tended to clog the nozzle and overprint, while the 2 ME MA MeSDVC and the GM printed with the best shape fidelity. Scale bar: 5 mm. Different letters indicate significance from each other (*p* < 0.05). ns = no significance across all groups.

The precursor rheology was characterized by a yield stress test (Figure 5a) with PF-127 and unfunctionalized SDVC included as comparator groups. Given that PF-127 is a commonly used printable support material, rheology on PF-127 was included as a reference point. PF-127 had a yield stress of 1360 ± 170 Pa, which was 1.6 to 33.2-times greater than those of all other groups (*p* < 0.0001). The 2 ME precursor had a yield stress (850 ± 150 Pa) that was 1.75-, 2.2-, 2.0-, and 20.8-times greater than those of the 5 ME, 10 ME, GM, and SDVC precursors, respectively (*p* < 0.001). The 5 ME, 10 ME, and GM had yield stresses 9.4 to 11.9-times greater than that of the SDVC precursor (*p* < 0.01). Notably, the 20 ME precursor did not have a detectable yield stress.

The storage modulus recovery was measured ~5 s after completion of the high shear step, and all hydrogel precursors, except the 20 ME and SDVC, recovered a storage modulus within 5 s. For the SDVC precursor, the loss modulus was higher than the storage modulus immediately after the high shearing stopped; therefore, there was no immediate storage modulus recovery. The SDVC precursor did eventually recover a storage modulus, after 28 ± 15 s (data not shown), but not by the 5 s time point for measurement. The 20 ME precursor did not have a yield stress; therefore, storage modulus recovery was not tested. The storage modulus recoveries (Figure 5b) across all measurable MeSDVC precursors and PF-127 were not significantly different from each other (ranging from 46 ± 7% to 60 ± 9% recovery, with PF-127 having a recovery of 59 ± 14%).

From the viscosity curves (Figure 5c), all groups were shear-thinning and compared to PF-127, which had a viscosity three to four orders of magnitude greater than those of all MeSDVC precursors and SDVC (*p* < 0.0001). The 2 ME precursors had 1.61-, 2.4-, 8.6 × 10^3^-, 2.5-, and 15-times greater viscosity than those of the 5 ME, 10 ME, 20 ME, GM precursors, and SDVC, respectively (*p* < 0.0001). The 5 ME precursor had 5.3 × 10^3^-, 1.55-, and 9.3-times greater viscosity than those of the 20 ME and GM precursors, and SDVC, respectively (*p* < 0.05). The 10 ME and GM precursor had 3.5 × 10^3^- and 6-times greater viscosities than those of the 20 ME precursor and SDVC, respectively (*p* < 0.01).

Images of all the MeSDVC batches, SDVC, and PF-127 precursors were taken (Figure 5d, top row). All batches of MeSDVC, except the 20 ME made with MA, were paste-like in consistency and held their shape on the spatula, which was similar to the paste-like nature of the PF-127. Interestingly, the 20 ME made with MA was not paste-like in consistency and was not printable.

### 3.4. Printability Characterization

Each MeSDVC precursor, SDVC, and PF-127 were printed into a four-layer grid (Figure 5d, bottom row). PF-127 was printed with distinct struts and layers with a pore area of 0.60 ± 0.08 mm^2^ and strut size of 0.67 ± 0.02 mm. The pore area of the PF-127 was 1.9 to 5.1 times greater than those of all other printed MeSDVC precursors. There were no significant differences in strut sizes among any of the groups. SDVC was printed for comparison of the materials prior to functionalization. Each printed strut of SDVC immediately relaxed and closed all the pores (no measurable pore areas or strut sizes). From the side view, the printed layers of the SDVC were not distinguishable. The 2 ME MeSDVC printed with minimal clogging; each layer was visible from the side. The pore area was 0.32 ± 0.2 mm^2^ and the strut size was 0.89 ± 0.18 mm. The 5 ME and 10 ME MeSDVC precursors required higher pressures to extrude (~10.8–12.2 psig) than the 2 ME (~8–9.5 psig) and had increased clogging, which led to overprinting. The 5 ME and 10 ME MeSDVC precursors did not print consistently, and the pore area/strut size was not able to be consistently controlled by altering the pressure, unlike what was possible with the 2 ME. Of the fewer good prints, the 5 ME and 10 ME precursors both had pore areas of 0.18 ± 0.13 mm^2^ and strut sizes of 0.92 ± 0.18 and 0.80 ± 0.14 mm, respectively. The 20 ME MeSDVC precursor was not paste-like in consistency and was not printable. The GM MeSDVC precursor printed at higher pressures (13.2 psi) than the 2 ME MeSDVC precursor (8–10 psi) and had minimal clogging. The GM MeSDVC had a pore area of 0.12 ± 0.10 mm^2^ and strut size of 0.92 ± 0.12 mm.

**Figure 6:**
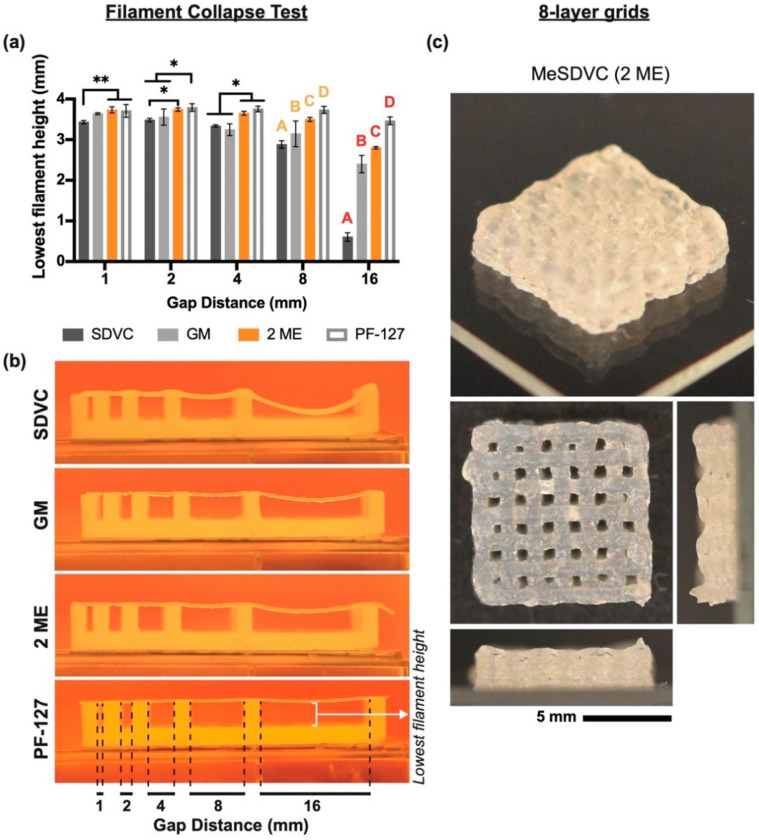
The 2 ME MA MeSDVC had the best printability and ability to print taller structures. (**a**,**b**) The filament collapse test consisted of material precursors being printed over gaps of different sizes (i.e., 1, 2, 4, 8, and 16 mm) and modified analyses were performed, where the lowest filament height between the gaps was measured (n = 3–6). The 2 ME MA MeSDVC and the GM MeSDVC were tested, given that the 5 ME and 10 ME were not able to complete the test due to nozzle clogging. SDVC and PF-127 were included as comparators. Overall, the 2 ME MA MeSDVC precursor was able to span larger gaps with less collapse than the GM MeSDVC precursor and SDVC. (**c**) The 2 ME MA MeSDVC precursor was printed in an eight-layer grid to show the ability to print taller scaffolds without good shape fidelity and without collapse. * *p* < 0.05. ** *p* < 0.01. Letters indicate significance from other letters of the same color (*p* < 0.05). Scale bar: 5 mm.

To characterize the printability of the MeSDVC precursors, the filament collapse test was performed and a modified analysis of the lowest filament height was performed (Figure 6a,b). The 5 ME and 10 ME precursors were not able to print all the way across all the gaps without breaking or clogging and were therefore not included in the analysis. For the 16-mm gap distance, the PF-127 filament height (3.46 ± 0.09 mm) was 1.2 to 5.7 times greater than those of the 2 ME precursor, GM precursor, and SDVC (*p* < 0.0001). The 2 ME precursor filament height (2.80 ± 0.03 mm) was 0.2 to 4.6 times greater than those of the GM precursor and SDVC, respectively (*p* < 0.001). The GM precursor filament height (2.40 ± 0.2 mm) was four times greater than that of the SDVC (*p* < 0.0001). For the 8-mm gap distance, the PF-127 filament height (3.73 ± 0.09 mm) was 7% to 29% greater than those of the 2 ME precursor, GM precursor, and SDVC (*p* < 0.05). The 2 ME precursor filament height (3.50 ± 0.05 mm) was 11% and 21% greater than those of the GM precursor and SDVC, respectively (*p* < 0.01). The GM precursor filament height (3.14 ± 0.3 mm) was 9% greater than that of the SDVC (2.88 ± 0.09 mm) (*p* < 0.05). To demonstrate the ability of the best-performing bioink to print taller structures, an eight-layer grid was printed with the 2 ME MeSDVC precursor (Figure 6c). The pore areas were 0.25 ± 0.11 mm^2^ and the individual layers were distinguishable from each other.

### 3.5. Printed MeSDVC In Vitro Cell Viability Study

**Figure 7:**
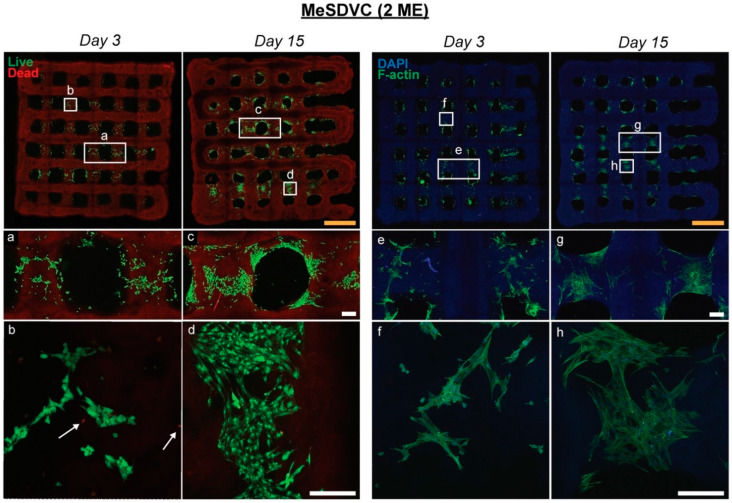
The printed 2 ME MA MeSDVC hydrogels supported rat bone marrow-derived mesenchymal stem cell (rBMSC) viability and adhesion over 15 days. The 2 ME MA MeSDVC was printed into four-layer grids, crosslinked with UV light, and rBMSCs were seeded onto the scaffolds for 15 days (n = 3). Live/dead staining (left) and F-actin/DAPI staining (right) were performed after 3 and 15 days of culture to assess viability and adhesion. The printed struts were visible from autofluorescence in the dead and DAPI channel (top row). At both time points, rBMSCs exhibited high viability and limited cell death (dead cells indicated by white arrows in insets b and d). From the F-actin and DAPI staining, after 3 days, cells were distributed loosely around the struts in comparison to after 15 days, where cells were tightly adhered around the printed strut and had increased spreading. Yellow scale bars: 2 mm. White scale bars: 200 µm.

The printed 2 ME MeSDVC hydrogel supported the viability and adhesion of seeded rBMSCs (Figure 7). The MeSDVC printed struts were visible from autofluorescence in the dead channel and the DAPI channel. The rBMSCs were not visible on the top layer of printed struts; however, rBMSCs had settled and grew on struts of the layer underneath the top printed layer, as seen from the cell distribution after 3 and 15 days of culture (Figure 7, top row). From LIVE/DEAD and F-actin fluorescent staining after 3 days of culture, the rBMSCs were distributed loosely around the struts (Figure 7, insets a, e), in comparison to after 15 days, where the rBMSCs tightly adhered to the struts (Figure 7, insets c, g). LIVE/DEAD staining (Figure 7, left) showed live rBMSCs and few dead cells (Figure 7, insets b, d, indicated by a white arrow). F-actin fluorescent staining showed rBMSCs spreading and adhering to the printed struts after 3 days (Figure 7, inset f) and increased spreading after 15 days (Figure 7, inset h).

## 4. Discussion

In the current study, we investigated a well-known methacryloyl-modifying reagent, methacrylic anhydride, for functionalizing solubilized cartilage matrix and fabricating a cartilage-only hydrogel with the stiffness approaching that of native cartilage and a paste-like precursor for both easy surgical placement and bioprinting applications. Using a 2-fold molar excess of MA, the resulting cartilage hydrogel, MeSDVC, had (1) the highest stiffness of any cartilage-only hydrogel fabricated to date, which mimicked native cartilage, and (2) had paste-like precursor rheology, which produced a promising printable biomaterial and may facilitate future surgical delivery in patients.

In the few studies with methacryl-modified cartilage matrix, and across methacryl-modified gelatin studies in general, it is most common to qualitatively confirm functionalization with NMR [2,17,25] or quantify functionalization by measuring the decrease in amino groups with NMR or the 2,4,6-trinitrobenzene-sulfonic acid (TNBS) [23] assay. However, measuring the decrease in amino groups only quantifies amino groups that obtain functionalized (methacrylamides) and ignores the hydroxyl groups that obtain functionalized (methacrylates). Therefore, in the current study, we used NMR with an internal standard (i.e., TMSP) to quantify the methacrylates and total methacryloyls in mmol/g of material, and taking the difference to calculate the methacrylamide content, which was done before for methacryl-modified gelatin by Claaßen et al. [31]. In the current study, the MA at all molar excesses tested (2–20 ME) resulted in higher total methacryloylation of cartilage matrix than the highest molar excess of GM (20 ME). While the MA was able to functionalize both hydroxyl and amino groups, the GM appeared to mainly functionalize hydroxyl groups, which has been noted by others [38,39]. As the molar excess of MA increased, the total methacryloylation of MeSDVC increased, which was not surprising. While the amount of methacrylate increased with increased MA, the methacrylamide content remained the same across the different molar excesses of MA used, most likely because the amino groups have a higher reactivity to MA than hydroxyl groups. Claaßen et al. [31]. observed the same methacrylate/methacrylamide trends when using different molar excesses of MA to functionalize gelatin. The total methacryloylation achieved with MA in the current study (0.2–1.08 mmol/g) was similar to those achieved with MA-modified gelatin (0.34–0.96 mmol/g) [31], although the amounts of MA used during the synthesis of MeSDVC in the current study were higher than those used by Claaßen et al.

The fluid mechanics of the precursor are important to characterize and refine to improve surgical placement of the implanted material and, additionally, are critical for developing printable biomaterials for the bioprinting field [35]. We characterized three rheological properties: yield stress, storage modulus recovery, and viscosity. We previously found an upper limit of ~1000 Pa for high molecular weight (i.e., 1 and 1.5 MDa) hyaluronic acid hydrogels [34]; however, for the cartilage matrix-based materials, we now recommend that the yield stress be between ~100 and 2000 Pa to be high enough to hold its shape after implantation or bioprinting, but not putty-like and difficult to spread/manipulate. All the fabricated MeSDVC materials (except the 20 ME MA MeSDVC) had a yield stress within this updated range and had acceptable paste-like qualities for implanting within a defect. The yield stress of the GM MeSDVC precursor in the current study (420 ± 70 Pa) was about half of the yield stress of the GM MeSDVC precursor in previous studies (~750 Pa) [25] and in the range of the 5 and 10 ME MA MeSDVC precursors. Interestingly, the 20 ME MeSDVC in the current study was liquid-like with no detectable yield stress and was not suitable for extrusion printing. Consequently, the 20 ME MeSDVC may not be suitable for implanting into a cartilage defect (i.e., can leak out before crosslinking). However, it is worthwhile to note that such low-viscosity precursors may be suitable for other types of bioprinting (e.g., digital light processing, inkjet) [40,41].

In addition to having a yield stress, the storage modulus of a printable biomaterial or an implantable hydrogel precursor needs to recover quickly after experiencing shear forces (i.e., such as those experienced from extrusion from a syringe during implantation or printing), and the exact percentage required depends on the initial storage modulus. Materials with higher yield stresses can have lower recoveries and still print with acceptable shape fidelity, while materials with lower yield stresses may need higher recoveries to maintain shape after extrusion. For example, in the current study, the unfunctionalized SDVC possessed a low yield stress (41 ± 7 Pa); however, after shearing during the storage modulus recovery test, the loss modulus of the SDVC was higher than the storage modulus and it took 28 ± 15 s for the storage modulus to “recover” (i.e., surpass the loss modulus) after shearing. We anticipated that the liquid-like behavior of SDVC that dominated after shearing would lead to poor shape fidelity after being extruded. While the unfunctionalized SDVC may not be a viable printable biomaterial, all the functionalized MeSDVC materials (except the 20 ME MA MeSDVC) had high yield stresses (i.e., >390 Pa) and acceptable storage modulus recoveries from 46 to 60% and, therefore, had the potential to be printable.

The viscosity profiles of higher viscosity paste-like materials need to be shear thinning for successful extrusion through a syringe, for injecting into a defect, and for extrusion bioprinting. The precursor of the 20 ME MeSDVC had lower viscosities, but all other MeSDVC materials with higher viscosities were shear thinning and were able to extrude through a syringe with a 22 G tapered plastic nozzle, making them potentially printable. Few studies have characterized the rheology of hydrogel precursors except in bioprinting applications [21,40], and standardized rheological characterizations (i.e., yield stress, storage modulus recovery, and viscosity) are greatly needed in the cartilage matrix hydrogel literature for comparing and identifying materials suitable for surgical placement and bioprinting.

Utilizing the quantified functionalization via NMR, we found an interesting trend in MeSDVC precursor rheology with increased functionalization with MA. In terms of precursor rheology, the least functionalized MeSDVC (2 ME, 0.37 mmol/g) had the highest yield stress (850 ± 150 Pa) and viscosities, whereas the most functionalized MeSDVC (20 ME, 1.08 mmol/g) had the lowest viscosities and no detectable yield stress. The liquid precursor of the 20 ME MeSDVC was interesting because even the unfunctionalized SDVC had a low yield stress (41 ± 7 Pa). Given that the cartilage particles were pepsin-digested for 48 h at room temperature, we speculate that the native collagen content was most likely denatured (i.e., gelatin) and that there would be limited collagen fibrillation/rheological changes upon heating to 37 °C. We speculate that SDVC and low functionalized MeSDVC may be more similar to gelatin, and the denatured collagen strands were able to form partial triple helices (giving it a paste-like quality) at room temperature. Additionally, we speculate that the low functionalization MeSDVC was more viscous than the unfunctionalized SDVC because of increased branching and entanglements of the functional groups. We believe the SDVC only had partial helix formation, while the MeSDVC may have had synergy between greater entanglements of polymer chains from the methacryl modifications and increased helix formation because of the closer proximity of polymers. On the other hand, at higher functionalizations, we speculate that the methacryloyls may have interrupted collagen strands from forming triple helices, resulting in lower viscosities. Others have similarly observed decreased viscosity of gelatin at higher methacryloylation and even tailored the precursor viscosity by double functionalization with methacryloylation and acetylation to further decrease the viscosity, specifically to make suitable bioinks for inkjet bioprinting [40].

The methacryloylation of SDVC into MeSDVC not only made photocrosslinking possible but enhanced the rheology and printability of SDVC. Rheological and printing characterizations (i.e., printed grids, filament collapse test) were vital in identifying differences in printability based on the degree of functionalization. Unfunctionalized SDVC, which had a yield stress but no storage modulus recovery after shearing, was not suitable for printing given that printed struts were not able to maintain their shape, and the SDVC filament had the lowest height across the largest gap distances in the filament collapse test compared to the 2 ME and GM precursors. However, after methacryloyl modification, the MeSDVC 2 ME and GM were successfully printed with good shape fidelity, consistent pore/strut sizes, and had higher filament heights during the collapse test than the SDVC filament height. Comparing the MeSDVC 2ME and GM precursors, the modified filament collapse test distinguished that the 2 ME MeSDVC was able to better bridge larger gaps (i.e., greater lowest filament heights) and, therefore, had better printability than the GM MeSDVC.

Interestingly, while the 5 and 10 ME MeSDVC precursors did have a paste-like rheology, they were not suitable for printing. The 5 and 10 ME MeSDVC hydrogel precursors had tendencies to clog the 22 G nozzle and required higher pressures to print with than the 2 ME. Furthermore, the 5 and 10 ME MeSDVC precursors were not able to complete the filament collapse test due to the preliminary breakage of the filament or clogging. We speculate that there may have been heterogeneous functionalization leading to areas of lower functionalization/higher viscosities that may have caused increased clogging. Overall, the lowest methacryloylation of the MeSDVC (i.e., 2 ME) enabled the printability of the cartilage matrix as a stand-alone printable biomaterial; however, higher degrees of methacryloylation (i.e., 5 and 10 ME) resulted in nozzle clogging during printing, and the highest degree of methacryloylation (i.e., 20 ME) had too low of a viscosity and was not printable.

While the fluid mechanics of the hydrogel precursor are important for surgical placement and bioprinting, the solid mechanics and swelling of the crosslinked hydrogel are vital for material retention and performance in a cartilage defect. In the current study, the 2 ME MeSDVC had a compressive modulus of 1.55 ± 0.23 MPa, which is in the range of the native cartilage (~1.8 MPa) [2], higher than what we have previously achieved (~0.675–1 MPa) [2,25], and higher than any other cartilage matrix-based hydrogel with reported testing to date (not including native cartilage plugs or scaffolds reinforced with polymers). Chondral/osteochondral allografts and those with perforations retain the mechanical performance of the native cartilage, but may have challenges in surgical delivery (e.g., removal of healthy cartilage to fit the shape of the implant) and may be limited by donor supply. Breaking down the cartilage into fragments or particles and creating a scaffold may enable easier implantation into an irregularly-shaped cartilage defect. The majority of cartilage matrix-based scaffolds freeze-dry cartilage particle slurries and further crosslink them with DHT [9], DHT plus carbodiimide [11,14,18,42], glyoxal [10], UV [43], or genipin [12,19]. Others have incorporated cartilage particles or solubilized cartilage particles (e.g., with pepsin) into existing hydrogels (e.g., alginate [21], collagen I [44], GelMA [15,16]). Finally, a few studies methacryloylated the solubilized cartilage matrix, but incorporated it with other hydrogels (e.g., GelMA, HA + gelatin) [17,23]. Notably, all the aforementioned cartilage-based scaffolds had elastic moduli under 160 kPa. The only cartilage matrix scaffolds that have achieved elastic moduli greater than 1 MPa were by combining the cartilage matrix with a polymer-reinforced scaffold [21,22].

In the current study, with a methacryl-modified cartilage matrix alone, we achieved a hydrogel that is 10 times stiffer than the majority of hydrogels employed in regenerative medicine, and 1.5 times stiffer than even nanocomposite hydrogels, or hydrogels reinforced with nanomaterials (e.g., carbon-based, ceramic, metallic, polymeric), which typically have compressive elastic moduli less than 1 MPa [3,4,5,6,7,8]. In our previous studies with MeSDVC functionalized with GM, we achieved a stiffness of ~0.675–1 MPa [2,25]; however, our previous GM MeSDVC concentration was double our current study’s MA MeSDVC concentration (20 wt% versus 10 wt%). Additionally, the GM MeSDVC synthesized in the current study (and in our previous studies) used 10 times more of the methacrylating reagent than the MA MeSDVC in the current study (20-fold molar excess vs. a 2-fold molar excess). We speculate that the increased mechanical performance of our MA and GM-functionalized MeSDVC compared to our previous MeSDVC [45] was due to higher functionalization. Unfortunately, a limitation of our previous work is that we were not able to quantify the degree of functionalization, as we have done in the current studies. Therefore, we were unable to determine if the MeSDVC synthesized in the current study had higher functionalization than previous MeSDVCs and if that may have contributed to the enhanced mechanical performance. However, we are now quantifying functionalization via the inclusion of a TMSP standard [30,31] and will continue to use the TMSP method to quantify functionalization moving forward. We recommend for others in the field do so as well to enable a direct comparison of the degree of functionalization across any material or functionalization. Comparing our MeSDVC to other cartilage-based hydrogels, we speculate that the enhanced mechanical performance was from higher matrix concentrations (i.e., 10 wt%) and/or higher functionalization, given that typically used matrix concentrations are below 5 wt%. We found in previous studies [25] and with the current 20-ME MA MeSDVC hydrogels that doubling the matrix concentration from 10 to 20 wt% can result in a 1.9- to 5-fold increase in the compressive modulus (data not shown). While we cannot directly compare the degree of functionalization of our MeSDVC to other studies in the literature, one distinct advantage of the TMSP quantification of functionalization is that we will be able to directly compare the degree of functionalization of our MeSDVC to any future material quantified with the same method.

Finally, the failure strain of the decellularized version of MeSDVC, which was called MeSDCC (20 wt%), was low in our previous studies (~7.5% strain) [2], and previous MeSDVC (10 wt%) hydrogels were only tested up to a 10% strain [45]. In the current study, we were able to reach at least a 20% strain without failure. While we have not found differences in total GAG or collagen content between MeSDVC and MeSDCC [45], the decellularization process may have compromised the structural integrity of the ECM, thereby reducing the mechanical performance [46]. One limitation of the current study was not testing to failure to determine the failure strain. An interesting future avenue to further increase the mechanical performance in terms of the fracture strain, tensile strength, and flexibility may include the addition of nanomaterials to form a nanocomposite hydrogel [47,48] to leverage the reversible physical interactions between nanoparticles and the polymers to dissipate stress. In the future, implantation of the developed mechanically robust cartilage-based hydrogel may enable patients to be weight-bearing immediately after being implanted, which may improve cartilage repair [49], instead of being limited to non-weight-bearing activities for 6 weeks or more.

With the quantification of functionalization, we found another interesting trend in hydrogel stiffness with increased functionalization. The least functionalized MA MeSDVC hydrogels (i.e., 2, 5 ME) were stiffer than the more functionalized MA MeSDVC hydrogels (i.e., 10, 20 ME). We do not believe there was a lower degree of crosslinking in the higher functionalized MeSDVC hydrogels based on the absorption and swelling, which were generally lower for higher functionalized MeSDVC (lower swelling indicated a greater degree of crosslinking). Given that the less functionalized MeSDVC precursor had more physical interactions (demonstrated by the paste-like rheology) between the polymer chains, we speculate that the initial physical interactions led to more efficient UV crosslinking compared to the higher functionalized MeSDVC that had a liquid precursor and possibly fewer physical polymer entanglements. Hoorick et al. [50] similarly noted that modified gelatin had a higher modulus when UV crosslinked as a physical gel at 5 °C, compared to when UV crosslinked as a liquid at 37 °C. The authors speculated that the UV crosslinking was more efficient when the gelatin had pre-formed triple helices, compared to when triple helices were not induced. Given that collagen II is a major component of the cartilage matrix, we speculate the same theory may apply to MeSDVC. Alternatively, aggrecan may have been functionalized, as it consists of glycosaminoglycans (GAGs), chondroitin, and keratan sulfates, which have hydroxyls that can be functionalized. Given that the strong anionic charges of the GAG chains contribute greatly to the compressive strength of native cartilage [51], we speculate that high functionalization of aggrecan/GAG hydroxyls may have inhibited the anionic charge of the GAG chains, therefore decreasing the compressive strength.

In addition to the mechanical performance of an implanted scaffold, the swelling characteristics of hydrogels can affect the retention of the material within the defect. Depending on the polymer, some hydrogels can swell out or contract after fabrication, which may lead to dislodgment or poor integration with the existing tissue after in situ gelation. Collagen hydrogels are known for contracting [52,53] and given the high collagen content of cartilage matrix, the swelling and absorption of water of cartilage matrix-based hydrogels is a vitally important parameter. All the MeSDVC hydrogels (10 wt%) in the current study had similar swelling ratios to the 10 wt% MeSDVC hydrogels (~10) we previously characterized [25]. However, these swelling ratios do not reflect the mass of water taken up after the fabrication of the hydrogel. Given the need to characterize the amount of water uptake from in situ fabrication to an equilibrium swollen state, another measure of swelling (absorption) is important to characterize, where absorption is the swollen mass divided by the fabricated mass (i.e., hydrogel mass immediately after fabrication). The absorption ratio indicates whether the material will swell (>1), contract (<1), or ideally do neither and stay the same size as originally fabricated (=1). Fortunately, both the GM and all the MA MeSDVC hydrogels had absorptions close to 1 (0.84 ± 0.03–1.26 ± 0.05), which led to <1% changes in the hydrogel diameters after swelling. The minimal water uptake/changes in the diameter of the MeSDVC hydrogel are promising for the retainment of the hydrogel within a cartilage defect, preventing the hydrogel from swelling out of the defect and from immediately contracting and, thus, avoiding potential poor tissue integration.

To ensure that the high functionalization of MeSDVC did not disrupt cell adhesion or reduce viability, the printed and crosslinked 2 ME MeSDVC hydrogels were seeded with rBMSCs and cultured for 15 days. After 3 days, the rBMSCs had good viability and F-actin staining confirmed that rBMSCs loosely adhered around the second layer of printed struts (from the top). After 15 days of culture, the rBMSCs maintained good viability and the hydrogels were structurally intact (as compared to gelatin hydrogels, which can degrade in less than 2 weeks). F-actin staining showed increased spreading of the rBMSCs on the printed struts and was more tightly adhered to the struts compared to day 3. Given that the cells had settled on the struts of the second layer and not the top layer, future studies may include adjusting the pore size and/or print path to improve seeding homogeneity across the entire scaffold. Cell encapsulation in MeSDVC as a true bioink may additionally overcome the problems with heterogeneous cell seeding. The good printability and biocompatibility of MeSDVC combined with biocompatible photocrosslinking processes make MeSDVC a promising future bioink with encapsulated and printed cells. The rBMSCs have shown high viability after encapsulation, bioprinting, and UV crosslinking within printed MeSDVC scaffolds (data not shown) and will be the focus of future work.

In addition to biocompatibility with BMSCs, which are the most accessible cell sources (via microfracture), MeSDVC as a cartilage-based biomaterial may have potential chondroinductivity, which may result in more hyaline-like cartilage repair [1,25]. While we anticipate that the MeSDVC material alone may not be robustly chondroinductive, the addition of DVC or chondrogenic growth factors (e.g., TGF-β3) has synergistically supported chondrogenesis [21,25,54]. For example, in a previous study, we found that MeSDVC with added DVC particles supported greater chondrogenic gene expression (i.e., SOX-9, ACAN, and COL II) in encapsulated rBMSCs compared to that in MeSDVC hydrogels alone after 2 and 3 weeks of culture [4]. We speculate that the solubilization and functionalization process of synthesizing MeSDVC may diminish bioactive factors present in native cartilage, thereby reducing the chondroinductivity. However, the addition of DVC particles, which are minimally processed and similar to native cartilage, may retain bioactive factors from native cartilage and may be responsible for the improved chondroinductivity of MeSDVC plus DVC hydrogels. The focus of the current studies was to improve the mechanical performance of the MeSDVC hydrogels, and future work will focus on confirming or enhancing the chondroinductivity. Overall, the enhanced mechanical performance and potential chondroinductivity make MeSDVC-based hydrogels promising for future in vivo cartilage repair.

## 5. Conclusions

In the current study, we evaluated two different methacryloylating reagents (methacrylic anhydride (MA) versus glycidyl methacrylate (GM)), at varying concentrations, for their ability to functionalize solubilized cartilage matrix and improve our photocrosslinking cartilage matrix hydrogel, MeSDVC. We found that the paste-like precursor of the MeSDVC made with a 2-fold molar excess of MA had the highest stiffness and was the most printable. We made the stiffest cartilage matrix-based hydrogel (with the same stiffness as native cartilage) that does not use added polymers, plastics, or nanomaterials for reinforcement. Furthermore, the MeSDVC has a paste-like precursor that prints with great shape fidelity and does not require added polymers or plastics for successful printing. In addition to functioning as a new printable biomaterial and potential future bioink, the precursor rheology of the developed MeSDVC has high clinical relevance for easy surgical delivery into a cartilage defect and the high stiffness/limited swelling may enable better retainment and integration into the surrounding cartilage. With the known potential chondroinductivity from the cartilage matrix combined with the enhanced translation to the clinic shown in the current study, MeSDVC hydrogels have the foundation for becoming a clinical cartilage treatment.

## Figures and Tables

**Table 1:** Printing parameters used on the BioAssemblyBot for each hydrogel precursor.

	PF-127	SDVC	MeSDVC MA 2 ME	MeSDVC MA 5 ME	MeSDVC MA 10 ME	MeSDVC MA 20 ME	MeSDVC GM 20 ME
Tip size/style	22 G/tapered nozzle					Not printable	22 G/ tapered nozzle
Print speed (mm/s)	8						8
Print pressure (psig)	13	3	8–10	12	9.2		13.2
Start delay (ms)	50	25	250	50	200		25
Line width (mm)	0.25						0.25
Line height (mm)	0.25						0.25

## Data Availability

The data presented in this study are available upon request from the corresponding author.
